# Taking care of a diarrhea epidemic in an urban hospital in Bangladesh: Appraisal of putative causes, presentation, management, and deaths averted

**DOI:** 10.1371/journal.pntd.0009953

**Published:** 2021-11-15

**Authors:** S. M. Tafsir Hasan, Subhasish Das, A. S. G. Faruque, Azharul Islam Khan, John D. Clemens, Tahmeed Ahmed

**Affiliations:** 1 Nutrition and Clinical Services Division, International Centre for Diarrhoeal Disease Research, Bangladesh (icddr,b), Dhaka, Bangladesh; 2 Office of the Executive Director, International Centre for Diarrhoeal Disease Research, Bangladesh (icddr,b), Dhaka, Bangladesh; Lowell General Hospital, UNITED STATES

## Abstract

**Background:**

In April 2018, a diarrhea epidemic broke out in Dhaka city and adjoining areas, which continued through May. The Dhaka Hospital of the International Centre for Diarrhoeal Disease Research, Bangladesh (icddr,b), a dedicated diarrheal disease hospital, had a large upsurge in patient visits during the epidemic. An enhanced understanding of the epidemiology of this epidemic may help health-related professionals better prepare for such events in the future. This study examined the microbial etiology and non-pathogen factors associated with diarrhea during the epidemic. The study also evaluated the patients’ presentation and clinical course and estimated the potential mortality averted by treating patients during the epidemic.

**Methodology/Principal findings:**

Data from the patients who were treated at Dhaka Hospital during the diarrhea epidemic between April 2 and May 12, 2018 and were enrolled into the Diarrheal Disease Surveillance System (DDSS) at icddr,b were compared with the DDSS-enrolled patients treated during the seasonally-matched periods in the flanking years using logistic regression. icddr,b Dhaka Hospital treated 29,212 diarrheal patients during the 2018 epidemic period (and 25,950 patients per comparison period on average). *Vibrio cholerae* was the most common pathogen isolated (7,946 patients; 27%) and associated with diarrhea during the epidemic (adjusted odds ratio [AOR] 1.5, 95% CI: 1.1–2.0). The interaction of *Vibrio cholerae* with ETEC (AOR 2.7, 95% CI: 1.3–5.9) or *Campylobacter* (AOR 2.4, 95% CI: 1.1–5.1) was associated with further increased odds of diarrhea during the epidemic. In children under five years old, rotavirus was the most common pathogen (2,029 patients; 26%). Those who were adolescents (AOR 2.0, 95% CI: 1.3–3.1) and young adults (AOR 1.9, 95% CI: 1.4–2.5) compared to children younger than five years, resided within a 10 km radius of Dhaka Hospital (AOR 1.6, 95% CI: 1.1–2.2) compared to those living outside 20 km, borrowed money or relied on aid to pay for the transport to the hospital (AOR 1.6, 95% CI: 1.2–2.0), used tap water (AOR 1.8, 95% CI: 1.4–2.4) for drinking compared to tubewell water, and disposed of the solid waste directly outside the house (AOR 4.0, 95% CI: 2.7–5.9) were more likely to present with diarrhea during the epidemic. During the epidemic, patients were more likely to present with severe dehydration (odds ratio [OR] 1.6, 95% CI: 1.3–2.0) and require inpatient admission (OR 2.5, 95% CI: 1.9–3.3), intravenous rehydration (OR 1.7, 95% CI: 1.4–2.1), and antibiotics (OR 2.2, 95% CI: 1.8–2.7). The in-hospital case fatality rate was low (13 patients; 0.04%), and the hospital averted between 12,523 and 17,265 deaths during the epidemic.

**Conclusions/Significance:**

*Vibrio cholerae* played the primary role in the 2018 diarrhea epidemic in Dhaka. *Campylobacter*, enterotoxigenic *Escherichia coli*, and rotavirus had a secondary role. Adolescents and adults, residents of the metropolitan area, and those who were relatively poor and lacked safe water, sanitation, and hygiene (WASH) practices comprised the most vulnerable groups. Despite the increased disease severity during the epidemic, the case fatality rate was less than 0.1%. icddr,b Dhaka Hospital saved as many as 17,265 lives during the epidemic.

## Author summary

We analyzed records of patients presenting during a diarrhea epidemic in 2018 to the world’s largest diarrhea treatment hospital in Dhaka, Bangladesh. Patients presenting during the epidemic and the non-epidemic periods were compared at multiple levels. We found that *Vibrio cholerae* played a crucial role in the diarrhea epidemic. *Campylobacter*, enterotoxigenic *Escherichia coli*, and rotavirus had a secondary role. Adolescents and adults, residents of the Dhaka metropolitan area, and those who were relatively poor and lacked safe water, sanitation, and hygiene (WASH) practices were more vulnerable to the epidemic. Patients had increased disease severity during the epidemic, but the death rate was extremely low in the hospital. icddr,b Dhaka Hospital saved several thousand lives by taking care of diarrhea patients during the epidemic.

## Introduction

Diarrheal diseases are a common public health problem affecting all the continents, especially leading to increased mortality and morbidity in low- and middle-income countries (LMICs) [1]. In 2017, diarrheal illnesses were the eighth leading cause of mortality among all ages, responsible for about 1.6 million deaths. More than 87% of these deaths occurred in South Asia and sub-Saharan Africa, and more than one-third of the deaths occurred among children under five years old [2].

Diarrhea is caused by a wide array of viruses, bacteria, and parasites. However, the incidence and severity of diarrhea due to specific pathogens vary across age groups [1]. In 2017, while worldwide rotavirus was responsible for most deaths among children under five years old due to diarrhea, *Vibrio cholerae* was the leading cause of diarrhea mortality among adults [2]. Moreover, diversity in diarrheal morbidity, mortality, and microbial etiologies has been observed between high- and low-income countries [2]. Diarrheal diseases occur at a baseline frequency round the year and often follow a distinct seasonality in LMICs [3,4]. However, this disease endemicity may break out in epidemic proportions during natural disasters such as floods, cyclones, earthquakes, and conflicts. All large well-documented diarrhea epidemics in human history were due to *V*. *cholerae*, *Shigella*, or *Escherichia coli*. Epidemics can be extensive, and the attack rate, morbidity, and mortality are commonly much higher than the baseline [3,5–7].

Bangladesh is situated on the Ganges River Delta and prone to annual flooding in the monsoon. The country experiences a hot and humid summer from March to June, a warm and rainy monsoon from June to October, and a mild, dry winter from November to February [8,9]. Several common enteric pathogens causing diarrhea, including rotavirus, *Shigella*, *V*. *cholerae*, and *E*. *coli* are endemic in Bangladesh thanks to its floodplain topography and tropical monsoon climate that facilitate the pathogens’ replication and survival in the environment [10]. To make it worse, one-third of the urban population in this rapidly urbanizing country reside in crowded dwellings or slums lacking adequate safe water, sanitation, and hygiene [11]. It is presumed that all these factors are collectively responsible for the substantial burden of diarrheal illnesses in the country. In 2016, approximately 2.5 million diarrheal cases were reported in Bangladesh [12]. However, the actual diarrheal burden is even greater since many cases are managed at home with oral rehydration solution (ORS) and are left unreported [13].

The Dhaka Hospital of the International Centre for Diarrhoeal Disease Research, Bangladesh (icddr,b), located at the heart of Dhaka, the capital city of Bangladesh, is one of the largest dedicated diarrheal disease hospitals in the world. The hospital generally experiences biannual seasonal peaks of diarrheal cases–the summer peak predominantly associated with *V*. *cholerae* and enterotoxigenic *E*. *coli* (ETEC), and the winter peak mainly due to rotavirus. In April 2018, a diarrhea epidemic broke out in Dhaka city and adjoining areas, which continued through May. Dhaka Hospital had a large upsurge in patient visits during the epidemic, with a total visit of 40,950 diarrheal patients in April and May, which is 42% higher than the average number of visits during the same period in the past five years. Patient numbers exceeded the capacity of the hospital so greatly that it had to extend its services in a hurriedly built makeshift facility within the premises to take care of the extra patient load.

Although *V*. *cholerae* is endemic in Bangladesh, it is known to cause moderate to large diarrhea epidemics. However, cholera epidemics in the recent past were associated with major floods [14,15]. In contrast, the 2018 diarrhea epidemic took place in hot summer, clearly in the absence of any flooding or natural disasters. ETEC, in addition to *V*. *cholerae*, a common enteric pathogen causing diarrhea in Bangladesh, showed epidemic potential previously [15]. However, no in-depth formal investigations have been carried out to identify the pathogen(s) responsible, alone or in tandem, for the 2018 diarrhea epidemic.

An enhanced understanding of the epidemiology of the 2018 diarrhea epidemic and how Dhaka Hospital coped with the emergency may help physicians, nurses, public health experts, and health system professionals better prepare for such events in the future. In this study, we examined the microbial etiology and the non-pathogen factors (sociodemographic characteristics, water, sanitation, and hygiene practices, nutritional status, and health-related characteristics) associated with diarrhea during the epidemic among patients presenting to Dhaka Hospital. We evaluated the patients’ presentation and clinical course. Additionally, we assessed the role and effectiveness of the hospital in managing the epidemic by estimating the potential mortality it averted.

## Methods

### Ethics statement

The Research and Ethical Review Committees (Institutional Review Board) at the International Centre for Diarrhoeal Disease Research, Bangladesh (icddr,b) have approved the Diarrheal Disease Surveillance System (DDSS), its consent-taking procedures, and the policy for data preservation and use. In brief, on admission, verbal consent is sought from adult patients, or from adult guardians or caregivers on behalf of children or adults unable to provide consent. The Ethical Review Committee does not require a separate protocol to utilize and analyze anonymized DDSS data for research purposes. The details of the DDSS have been provided elsewhere [16].

### Study setting

Dhaka Hospital serves children and adults with diarrheal illnesses in inpatient and outpatient facilities. The hospital treats more than 150,000 cases of diarrhea annually. Diarrhea (passage of three or more loose stools in 24 hours) [17], with or without associated complications or comorbidities, is the criteria for admission into the hospital. The hospital imposes no restriction on the number of admissions, even during large surges. Dhaka Hospital has a regular capacity of 350 beds, which is expanded up to about 450 beds using additional mobile cholera cots, and even higher by erecting temporary tents if required. Interventions provided in the hospital for diarrhea include close monitoring of hydration status and administration of ORS, intravenous (IV) fluid, and use of antibiotics in selected cases. Patients receive additional care for comorbidities and complications of diarrhea, such as pneumonia, sepsis, malnutrition, and dyselectrolytemia. Comprehensive medical care, including laboratory investigations, medications, lodging, and food for both patients and their attendants, are provided free of cost.

The catchment area of the hospital consists of the densely populated metropolitan area of Dhaka, which has an estimated population of 15 million [18]. The majority of patients visiting the hospital are from socioeconomically disadvantaged communities and live in urban or peri-urban Dhaka. Tap water supplied by the water and sewerage authority is the primary source of water for the inhabitants of urban Dhaka, while people outside the metropolitan area frequently use tubewell water. The detailed description of icddr,b Dhaka Hospital has been reported previously [19].

### Diarrheal disease surveillance system

The Diarrheal Disease Surveillance System (DDSS) at icddr,b prospectively and routinely collects data on sociodemographic characteristics, water, sanitation, and hygiene (WASH) practices, enteric pathogens, nutritional status, and clinical presentation, management, and outcome from every 50^th^ patient treated at Dhaka Hospital [20]. A standardized questionnaire is administered to each patient to obtain the abovementioned information. Trained research assistants take anthropometric measurements at admission and discharge, using standard techniques and equipment [21,22]. A fresh stool sample is routinely collected from each DDSS-enrolled patient and tested for several high-priority [16] enteric pathogens, such as *V*. *cholerae*, ETEC, *Campylobacter* spp., *Aeromonas* spp., *Shigella* spp., non-typhoidal *Salmonella*, *and* rotavirus, applying standard laboratory methods that include culture, enzyme-linked immunosorbent assay (ELISA), and polymerase chain reaction (PCR) [23,24]. Samples were not routinely tested for other diarrheagenic *E*. *coli*, parasites, such as *Entamoeba histolytica*, *Giardia lamblia*, *Cryptosporidium*, or norovirus during the study period. All DDSS-related data are entered into an electronic database.

### Stool microbiology

After collection, fresh stool specimens from DDSS-enrolled patients were submitted to icddr,b central laboratories for routine screening of the aforementioned enteric pathogens. The details of the laboratory procedures for detecting enteropathogens in stool samples have been described elsewhere [16,19,25]. In brief, *Vibrio cholerae* were isolated by growth on tellurite taurocholate gelatin agar (TTGA) media with enrichment in bile peptone broth. Antisera panel testing (Denka Seiken Co., Ltd.) were performed for Ogawa or Inaba antigens. Phenotypic characterization (e.g., for El Tor and Classical) was done by 2.5% chicken cell agglutination tests. *Salmonella* spp. and *Shigella* spp. were isolated by growth on MacConkey agar and Salmonella-Shigella (SS) agar with enrichment in selenite broth followed by antisera panel testing (Denka Seiken Co., Ltd.). *Campylobacter* spp. was isolated by growth on Brucella agar. *Aeromonas* spp. were isolated by growth on TTGA and gelatin agar followed by phenotypic characterization of long-sugar metabolism.

For the detection of ETEC, fresh stool specimens were plated onto MacConkey agar. The plates were incubated at 37°C for 18 hours. Six lactose fermenting individual colonies morphologically resembling E. coli were isolated and tested for the presence of heat-stable toxin and heat-labile toxin using ganglioside GM1 ELISA and multiplex PCR [26,27]. The presence of Group A rotavirus-specific VP6 antigen in stool samples was detected by using the ProSpect Rotavirus kit (Oxoid Ltd, Basingstoke, UK), which utilizes a polyclonal antibody in a solid phase sandwich-type enzyme immunoassay according to the manufacturer’s instructions [28].

Resistance/susceptibility of *Vibrio cholerae* isolates to antimicrobials was determined by the disk diffusion method according to the guidelines of the Clinical Laboratory Standards Institute [29] with commercially available antimicrobial discs (Oxoid Ltd, Basingstoke, UK). The antibiotic discs used were azithromycin (15 μg), ciprofloxacin (5 μg), doxycycline (30 μg), erythromycin (15 μg), tetracycline (30 μg), and trimethoprim-sulfamethoxazole (25 μg).

### Study design and population

This study did a retrospective analysis of prospectively and routinely collected hospital surveillance (DDSS) data. An unmatched case-control design was considered to identify the pathogens and non-pathogen factors associated with diarrhea during the 2018 epidemic. Cases comprised all patients treated at Dhaka Hospital during the 2018 epidemic and enrolled into DDSS. Patients treated at the hospital during the seasonally matched periods in the flanking years and enrolled into DDSS were deemed controls. The study also compared the clinical features and outcomes of the cases with that of the controls.

### Definition of the epidemic

In this study, we defined the onset of the epidemic, following the method described by Schwartz et al. [14], as the first of three consecutive days in the summer of 2018 during which daily patient visits exceeded the 90^th^ percentile of daily visits in 2017 and 2019. The epidemic was considered to cease when the visits per day dropped below and never climbed back above the 90^th^ percentile for three consecutive days during the summer peak. The 90^th^ percentile for visits per day during 2017 and 2019 was 627. According to the definition stipulated, the 2018 diarrheal epidemic lasted from April 2 to May 12. Although the actual epidemic might have spanned beyond this period (either way), this objective definition ensured the identification of the peak period of the epidemic. The matching periods in the flanking years (April 2 to May 12 in 2017 and 2019) were considered as the comparator non-epidemic periods.

### Management of the epidemic

Dhaka Hospital is run by a team of experienced physicians, nurses, and support staff that managed diarrhea epidemics earlier [14,15]. To cope with the heavy patient load with increased severity during the 2018 diarrhea epidemic, the hospital had to reinforce its capacity. A makeshift ward with necessary facilities was built inside a tent erected on the icddr,b premises. Additional working hours were volunteered by regular hospital staff, including physicians and nurses. The hospital also borrowed (from research projects run by icddr,b) and hired additional workforces on a temporary basis. A constant supply of essential drugs, including ORS, IV fluids, antibiotics, and other hospital supplies, was ensured throughout the epidemic period.

### Data source and variables of interest

For this study, anonymized data on the study population were retrieved from the DDSS database. The number of daily patient visits and the list of deaths were obtained from the electronic hospital registry. Variables relevant to this analysis were carefully selected from among the variables available in the DDSS database (S1 Table). To examine the pathogens and non-pathogen factors associated with cases (compared to controls), data on variables indicating sociodemographic characteristics, WASH behavior, nutritional status and health, and enteric pathogens isolated from the study population were retrieved from the DDSS database.

Since income alone does not necessarily give an accurate impression of socioeconomic status [30,31], in addition to family income, we evaluated the highest education in the family [32] and whether the patient (or their family members) borrowed money or relied on aid to pay for the transport to the hospital [33,34]. We also constructed a wealth tertile variable by dividing a calculated household asset score into three quantiles. The household asset score was derived using principal component analysis (PCA) of the variables indicating possession of household assets. Variables included in the PCA were the patient’s housing condition (cemented floor, brick wall, concrete roof), the number of rooms in the household, access to electricity, access to gas for cooking, and ownership of a cot, an almirah, a fan, a radio, and a television.

The nutritional status was calculated for different age groups separately. For patients aged 0–4 years, thin (wasted), normal, and overweight were defined as weight for length/height Z score less than -2, between -2 and 2, and more than 2, respectively. For patients aged 5–19 years, thin, normal, and overweight were defined as body mass index (BMI) for age Z score less than -2, between -2 and 1, and more than 1, respectively. For patients aged 20 years and older, thin, normal, and overweight were defined as BMI less than 18.5, between 18.5 and 24.9, and 25 or higher, respectively. BMI was calculated by dividing the weight in kilograms by the height in meter squared. Patients’ weight measured at the time of discharge from the hospital was considered to calculate all weight-based anthropometric indices. The presence of edema was initially considered for assessing nutritional status, but no patients had bilateral pedal edema. Patients aged 0–19 years were identified as stunted if their length/height for age Z score was less than -2. The World Health Organization (WHO) standards and guidelines were used to calculate the Z scores and classify nutritional status, respectively [22,35,36].

To compare the and clinical presentation, management, and outcome between the cases and the controls, data on these variables during the epidemic and the comparison period were retrieved from the DDSS database or hospital registry (for total number of deaths).

### Statistical analysis

We visualized the weekly patient visits and the number of patients with major enteric pathogens detected in stool samples at Dhaka Hospital in 2018. Patients positive for selected enteric pathogens per week were estimated by multiplying the proportion of positive cases in DDSS samples by the actual figure for total patient visits obtained from the hospital registry.

We described the demographic characteristics, socioeconomic status, WASH behavior, nutritional status, and health-related characteristics of the DDSS-enrolled patients treated at Dhaka Hospital during the epidemic (cases) and the comparison period (controls) using frequency measures. We also described the distribution of the enteropathogens isolated from the cases and the controls, using frequency measures.

We evaluated the association of non-pathogen factors with diarrhea during the epidemic (cases) using simple and multivariable binomial logistic regression models. Variables with P < 0.2 in the unadjusted models were considered for multivariable model building [37]. However, the variables which were only available for a specific age group, such as taking vitamin A capsule in the past three months, were not considered for the multivariable model. The Bayesian Information Criterion (BIC) was a selection criterion for the final multivariable model [38], and all the variables in the final model had a P < 0.05. Multicollinearity was checked using the variance inflation factor (VIF). In the final model, the VIF for each variable was less than 2. The strength of association between a certain factor of interest and diarrhea during the epidemic was expressed as odds ratio (OR) and adjusted odds ratio (AOR) with 95% confidence interval (CI).

We examined the association of individual pathogens with diarrhea during the epidemic (cases) using simple and multivariable binomial logistic regression models. As multiple pathogens were isolated from some patients (coinfection), we also evaluated the presence of any pathogen-pathogen interactions (synergistic or multiplicative effects) in causing diarrhea during the epidemic using multivariable binomial logistic regression models. Since concurrent isolation of more than two pathogens from a single patient was rare, we explored all possible dual pathogen interactions. The statistical significance of the interaction terms was checked with the Wald test [39]. All the pathogen-related multivariable models were adjusted for the non-pathogen factors associated with diarrhea during the epidemic identified through another multivariable model described above. The strength of association between a certain pathogen (or interaction between two pathogens) and diarrhea during the epidemic was expressed as OR and AOR with 95% CI.

We visualized the percent distribution of the pathogens isolated from the cases and the controls by age groups. Percent distribution of types of *Vibrio cholerae* isolated from the two groups was visualized and Fisher’s exact test was used to compare the distribution between these two groups of patients. We described the antimicrobial resistance pattern of *Vibrio cholerae* isolated from the cases and the controls using frequency measures and compared it between the two groups using the chi-square test or Fisher’s exact test, as appropriate.

Hospital presentation, clinical course, and outcomes of the cases were compared with that of the controls using simple binomial logistic regression or Wilcoxon rank-sum test, as appropriate. The strength of association of a certain clinical feature or outcome with cases was expressed as OR with 95% CI. We visualized the relative effect of selective enteric pathogens on disease severity during the epidemic (among the cases) using a red-yellow-green color gradient. The association between a certain attribute indicating disease severity and an enteric pathogen was evaluated using binomial logistic regression, adjusted for age. To simplify the analysis, coinfections were ignored in the models. The strength of association was expressed as OR with 95% CI.

We adapted the method described by Oberle et al. to estimate the potential deaths averted by treating patients at the hospital during the epidemic [40]. To estimate the mortality averted, all DDSS based estimates were extrapolated to represent the total number of patients treated at the hospital during the epidemic. The estimations were done separately for each age group and then totaled. The minimum estimate (lower bound) of deaths averted was composed of 80% of all patients who survived after presenting with severe dehydration. The maximum estimate (upper bound) of deaths averted was composed of all patients who survived after presenting with severe dehydration, or with some or no dehydration but required IV rehydration later. The ‘mortality averted’ estimate is based on the early 20^th^ century observations when more than 90% of cholera patients with vascular collapse or severe dehydration would die since there was no effective rehydration therapy [41]. Dehydration status was assessed clinically by physicians or nurses using the WHO guidelines derived ‘Dhaka Method’ [5,16]. The clinical assessment of severe, some, and no dehydration approximate 10% or more, 5–9%, and less than 5% loss of body weight, respectively [16]. The proportion of potential deaths averted was estimated as follows: the number of deaths averted divided by the number of deaths averted plus the number of deaths observed. The proportion of patients that would have died without the hospital care was estimated as follows: the number of deaths averted divided by the number of patients treated at the hospital.

All statistical analyses were performed using Stata 15.1 (StataCorp, 4905 Lakeway Drive, College Station, Texas 77845 USA).

## Results

### Patient visits

**Fig 1:**
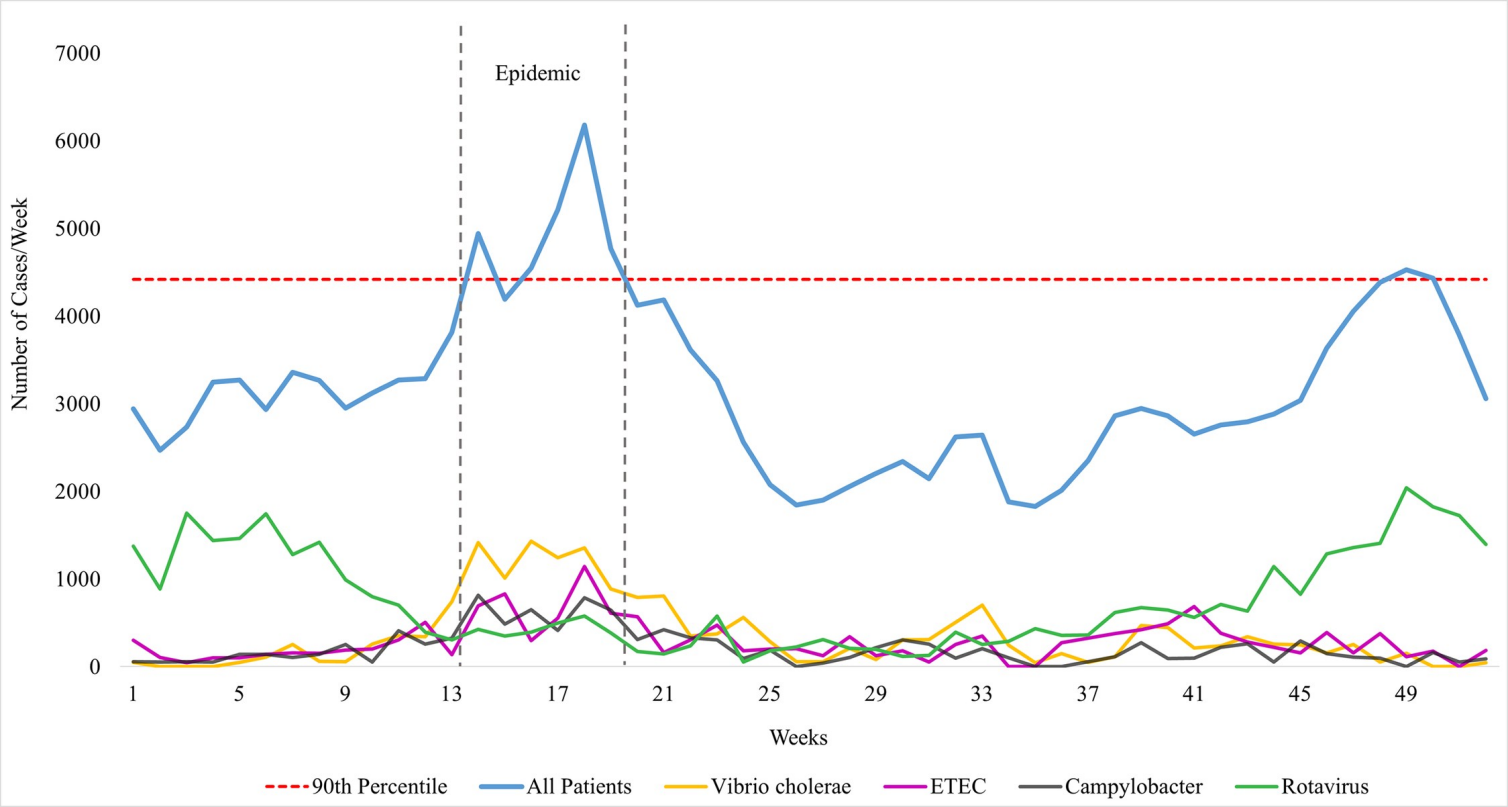
Total patient visits and patients with selected enteric pathogens detected in stool samples per week at icddr,b Dhaka Hospital in 2018. The horizontal dashed line represents the 90^th^ percentile of total patient visits per week in 2017 and 2019. The vertical dashed lines indicate the diarrhea epidemic period (April 2 to May 12) in 2018. ETEC, enterotoxigenic *Escherichia coli*.

The number of patients visiting Dhaka Hospital began to increase sharply during the last week of March 2018, reached an epidemic (as defined above) extent the following week, and remained elevated until the second week of May. Thereafter, patient visits gradually dropped to the pre-epidemic level over the next four weeks (Fig 1). Between April 2 and May 12, 2018, the period of epidemic identified in the study, Dhaka Hospital treated 29,212 diarrheal patients. The six-week-long epidemic peaked during the fifth week when the hospital received on average 883 patients a day. The lowest point in the epidemic was the second week, when the facility received about 599 cases per day. The hospital treated 51,900 patients altogether during the seasonally matched control periods in 2017 and 2019. A total of 562 and 969 patients were enrolled in the DDSS during the epidemic and the aggregate comparison period, respectively.

### Patient characteristics

Table 1 shows the characteristics of the patients presenting to Dhaka Hospital during the epidemic (cases) and the comparison period (controls). Patients aged 20 years or older constituted 61% of the cases and 51% of the seasonally matched controls. About 26% and 39% of the cases and the controls were children younger than five years, respectively. Males constituted 58% and 57% of the cases and the controls, respectively. Of the cases, 77% came from within a 20 km radius of Dhaka Hospital, and 68% of the controls came from this area. The absence of formal education in the family was found in 26% and 21% of the cases and the controls, respectively. About 13% of the cases and 14% of the controls had a monthly family income of less than USD 100. Tap water was the primary source of water for drinking among 78% of the cases and 57% of the seasonally matched controls. Of the patients, 99% reported using semi-sanitary/dug hole/open pit toilets. Most characteristics of the patients presenting during either of the individual comparator periods were similar to those observed for aggregate comparison period (S2 Table).

**Table 1:** Characteristics of DDSS-enrolled patients treated at icddr,b Dhaka Hospital during the epidemic (cases) and the comparison period (controls) and odds ratio of diarrhea during the epidemic (compared to the comparison period) for non-pathogen factors.

Characteristics	Cases (562), n (%)	Controls (969), n (%)	OR (95% CI)^a^	P	AOR (95% CI)^b^	P
**Demographic characteristics**						
Age, years						
0–4	148 (26.3)	380 (39.2)	ref	-	ref	-
5–9	13 (2.3)	23 (2.4)	1.5 (0.7–2.9)	0.301	1.2 (0.5–2.5)	0.702
10–19	57 (10.1)	68 (7.0)	2.2 (1.4–3.2)	<0.001	2.0 (1.3–3.1)	0.001
20–29	130 (23.1)	169 (17.4)	2.0 (1.5–2.7)	<0.001	1.9 (1.4–2.5)	<0.001
≥30	214 (38.1)	329 (34.0)	1.7 (1.3–2.2)	<0.001	1.4 (1.1–1.9)	0.008
Sex, male	328 (58.4)	551 (56.9)	1.1 (0.9–1.3)^c^	0.567		
Family members						
1–4	273 (48.6)	483 (49.9)	ref	-		
5–6	180 (32.0)	310 (32.0)	1.0 (0.8–1.3)	0.823		
≥7	109 (19.4)	176 (18.2)	1.1 (0.8–1.5)	0.524		
Place of residence^d^						
within 10 km radius of Dhaka Hospital	174 (31.0)	235 (24.3)	1.8 (1.4–2.4)	<0.001	1.6 (1.1–2.2)	0.008
within 10–20 km radius of Dhaka Hospital	260 (46.3)	420 (43.3)	1.5 (1.2–2.0)	0.001	1.1 (0.8–1.5)	0.466
outside 20 km radius of Dhaka Hospital	128 (22.8)	314 (32.4)	ref	-	ref	-
**Socioeconomic status**						
Highest education in the family, years						
none	144 (25.6)	207 (21.4)	1.5 (1.03–2.2)	0.036		
1–5	114 (20.3)	184 (19.0)	1.3 (0.9–2.0)	0.146		
6–10	133 (23.7)	281 (29.0)	1.0 (0.7–1.5)	0.912		
11–12	114 (20.3)	174 (18.0)	1.4 (1.0–2.1)	0.084		
>12	57 (10.1)	123 (12.7)	ref	-		
Family income, below USD 100 per month	73 (13.0)	137 (14.1)	0.9 (0.7–1.2)	0.529		
Wealth tertile						
low	186 (33.1)	332 (34.3)	1.0 (0.7–1.2)	0.739		
middle	194 (34.5)	326 (33.6)	1.0 (0.8–1.3)	0.898		
high	182 (32.4)	311 (32.1)	ref	-		
Reliance on aid or borrowing money for transport cost	169 (30.1)	190 (19.6)	1.8 (1.4–2.2)	<0.001	1.6 (1.2–2.0)	<0.001
**WASH behavior**						
Source of drinking water, tap water	437 (77.8)	549 (56.7)	2.7 (2.1–3.4)^e^	<0.001	1.8 (1.4–2.4)^d^	<0.001
Source of water for washing, tap water	438 (77.9)	558 (57.6)	2.6 (2.1–3.3)^e^	<0.001		
Frequency of water collection for drinking, ≤2 times per day	275 (48.9)	438 (45.2)	1.2 (0.9–1.4)	0.158		
Frequency of water collection for cooking, ≤2 times per day	445 (79.2)	720 (74.3)	1.3 (1.02–1.7)	0.031	1.4 (1.1–1.8)	0.014
Frequency of water collection for washing, ≤2 times per day	73 (13.0)	82 (8.5)	1.6 (1.2–2.3)	0.005	1.7 (1.2–2.5)	0.004
Treatment of water before drinking	299 (53.2)	547 (56.5)	0.9 (0.7–1.1)	0.218		
none	299 (53.2)	547 (56.5)	ref	-		
boiling	201 (35.8)	325 (33.5)	1.1 (0.9, 1.4)	0.283		
filter	60 (10.7)	88 (9.1)	1.2 (0.9, 1.8)	0.225		
other methods^f^	2 (0.4)	9 (0.9)	0.4 (0.1, 1.9)	0.252		
Use of sanitary toilet	4 (0.7)	12 (1.2)	0.6 (0.2–1.8)	0.335		
Disposal of solid waste directly outside the house	523 (93.1)	736 (76.0)	4.2 (3.0–6.1)	<0.001	4.0 (2.7–5.9)	<0.001
**Nutrition and health**						
Nutritional status^g^						
thin	117 (24.4)	173 (19.4)	1.4 (1.1–1.8)	0.016		
normal	296 (61.8)	611 (68.3)	ref	-		
overweight	66 (13.8)	110 (12.3)	1.2 (0.9–1.7)	0.211		
Stunted^g^ (aged 0–19 years)	43 (20.8)	83 (18.0)	1.2 (0.8–1.8)	0.405		
Took vitamin A capsule in past 3 months (aged 0–4 years)	52 (35.1)	181 (47.6)	0.6 (0.4–0.9)	0.010		
Recent measles (aged 0–4 years)	10 (6.8)	22 (5.8)	1.2 (0.5–2.6)	0.676		
Family members had diarrhea in the past week	65 (11.6)	122 (12.6)	0.9 (0.7–1.3)	0.555		

OR, odds ratio; AOR, adjusted odds ratio; CI, confidence interval; ref, reference.

^a^Odds ratio of diarrhea during the epidemic estimated from a simple binomial logistic regression model.

^b^Adjusted odds ratio of diarrhea during the epidemic estimated from a multivariable logistic regression model that includes age, place of residence relative to the location of Dhaka Hospital, whether the patient (or their family members) borrowed money or relied on aid to pay for the transport to the hospital, source of drinking water, frequency of collection of water for cooking and washing, and whether the patient’s family disposed of household solid waste directly outside the house.

^c^Reference = female

^d^Place of residence relative to the location of Dhaka Hospital

^e^Reference = tubewell water

^f^Other methods included alum, chlorine tablet, and sieving.

^g^Percentages calculated from non-missing values. Number of missing values: Nutritional status = 158, Stunted = 22.

### Non-pathogen factors associated with diarrhea during the epidemic

In unadjusted models, increasing age (10–19 years: OR 2.2, 95% CI 1.4–3.2; P < 0.001 and 20–29 years: OR 2.0, 95% CI 1.5–2.7; P < 0.001 and ≥30 years: OR 1.7, 95% CI 1.3–2.2; P < 0.001 compared to 0–4 years), residing within a 10 km radius of Dhaka Hospital (OR 1.8, 95% CI: 1.4–2.4; P = <0.001 compared to living outside 20 km), absence of formal education in the family (OR 1.5, 95% CI: 1.03–2.2; P = 0.036 compared to higher than 12 years of education), reliance on aid or borrowing money to pay for the transport to the hospital (OR 1.8, 95% CI: 1.4–2.2; P < 0.001), using tap water for drinking (OR 2.7, 95% CI: 2.1–3.4; P < 0.001) and washing (OR 2.6, 95% CI: 2.1–3.3; P < 0.001), collection of water no more than twice a day for cooking (OR 1.3, 95% CI: 1.02–1.7; P = 0.031) and washing (OR 1.6, 95% CI: 1.2–2.3; P = 0.005), disposal of the solid waste directly outside the house (OR 4.2, 95% CI: 3.0–6.1; P < 0.001), and thin nutritional status (OR 1.4, 95% CI: 1.1–1.8; P = 0.016) were associated with an increased odds of diarrhea during the epidemic compared to the comparison period. Among children younger than five years, receiving vitamin A capsule in the past three months was inversely associated with diarrhea during the epidemic (OR 0.6, 95% CI: 0.4–0.9; P = 0.010) (Table 1).

In the multivariable model, compared to children under five years old, adolescents (AOR 2.0, 95% CI: 1.3–3.1; P = 0.001), young adults (AOR 1.9, 95% CI: 1.4–2.5; P < 0.001) and adults (AOR 1.4, 95% CI: 1.1–1.9; P = 0.008) were more likely to present with diarrhea during the epidemic. Residing within a 10 km radius of Dhaka Hospital, compared to living outside 20 km, were associated with higher odds of diarrhea during the epidemic (AOR 1.6, 95% CI: 1.1–2.2; P = 0.008). Those who borrowed money or relied on aid to pay for the transport to the hospital were more likely to present with diarrhea during the epidemic (AOR 1.6, 95% CI: 1.2–2.0; P < 0.001). Compared to those who used tubewell water for drinking, patients drinking tap water had a higher occurrence of diarrhea during the epidemic (AOR 1.8, 95% CI: 1.4–2.4; P < 0.001). Those who collected water no more than twice a day for cooking (AOR 1.4, 95% CI: 1.1–1.8; P = 0.014) and washing (AOR 1.7, 95% CI: 1.2–2.5; P = 0.004) were more likely to present with diarrhea during the epidemic. People who disposed of the solid waste directly outside the house had higher odds of diarrhea during the epidemic (AOR 4.0, 95% CI: 2.7–5.9; P < 0.001) (Table 1).

### Microbial etiology

*V*. *cholerae* was the most common enteric pathogen, isolated from 27% and 17% of stool samples during the epidemic and the comparison period, respectively. Diarrhea of unknown etiology (no identifiable enteric pathogens) constituted 48% and 51% of the cases and the controls, respectively (Table 2).

**Table 2:** Distribution of enteric pathogens isolated from DDSS-enrolled patients treated at icddr,b Dhaka Hospital during the epidemic (cases) and the comparison period (controls) and odds ratio of diarrhea during the epidemic (compared to the comparison period) for high-priority pathogens.

Pathogen	Cases (562), n (%)	Controls (969), n (%)	OR (95% CI)^a^	P	AOR (95% CI)^b^	P
*Vibrio cholerae*	153 (27.2)	164 (16.9)	1.8 (1.4–2.4)	<0.001	1.5 (1.1–2.0)	0.004
ETEC	80 (14.2)	87 (9.0)	1.7 (1.2–2.3)	0.002	1.5 (1.1–2.2)	0.014
*Campylobacter* spp.	74 (13.2)	94 (9.7)	1.4 (1.02–2.0)	0.037	1.2 (0.9–1.7)	0.231
Rotavirus	44 (7.8)	84 (8.7)	0.9 (0.6–1.3)	0.567	1.6 (1.03–2.5)	0.035
*Aeromonas* spp.	33 (5.9)	123 (12.7)	0.4 (0.3–0.6)	<0.001	0.4 (0.3–0.6)	<0.001
*Shigella* spp.	9 (1.6)	28 (2.9)	0.5 (0.3–1.2)	0.119	0.5 (0.2–1.2)	0.135
NTS	7 (1.3)	8 (0.8)	1.5 (0.5–4.2)	0.425	1.1 (0.4–3.2)	0.840
Mixed pathogens (coinfection)^c^	93 (16.6)	105 (10.8)	1.6 (1.2, 2.2)	0.001	1.6 (1.1, 2.2)	0.007
Unknown etiology	271 (48.2)	491 (50.7)	0.9 (0.7, 1.1)	0.355	1.0 (0.8, 1.3)	0.963

OR, odds ratio; AOR, adjusted odds ratio; CI, confidence interval; ETEC, enterotoxigenic *Escherichia coli*; NTS, non-typhoidal *Salmonella*.

^a^Odds ratio of diarrhea during the epidemic estimated from a simple binomial logistic regression model.

^b^Adjusted odds ratio of diarrhea during the epidemic estimated from multivariable logistic regression models. Each pathogen model is adjusted for age, place of residence relative to the location of Dhaka Hospital, whether the patient (or their family members) borrowed money or relied on aid to pay for the transport to the hospital, source of drinking water, frequency of collection of water for cooking and washing, and whether the patient’s family disposed of household solid waste directly outside the house.

^c^For the mixed pathogens category, at least two pathogens were detected in the stool samples; however, 27 (1.8%) samples contained three pathogens, and 3 (0.2%) samples contained four pathogens.

**Fig 2:**
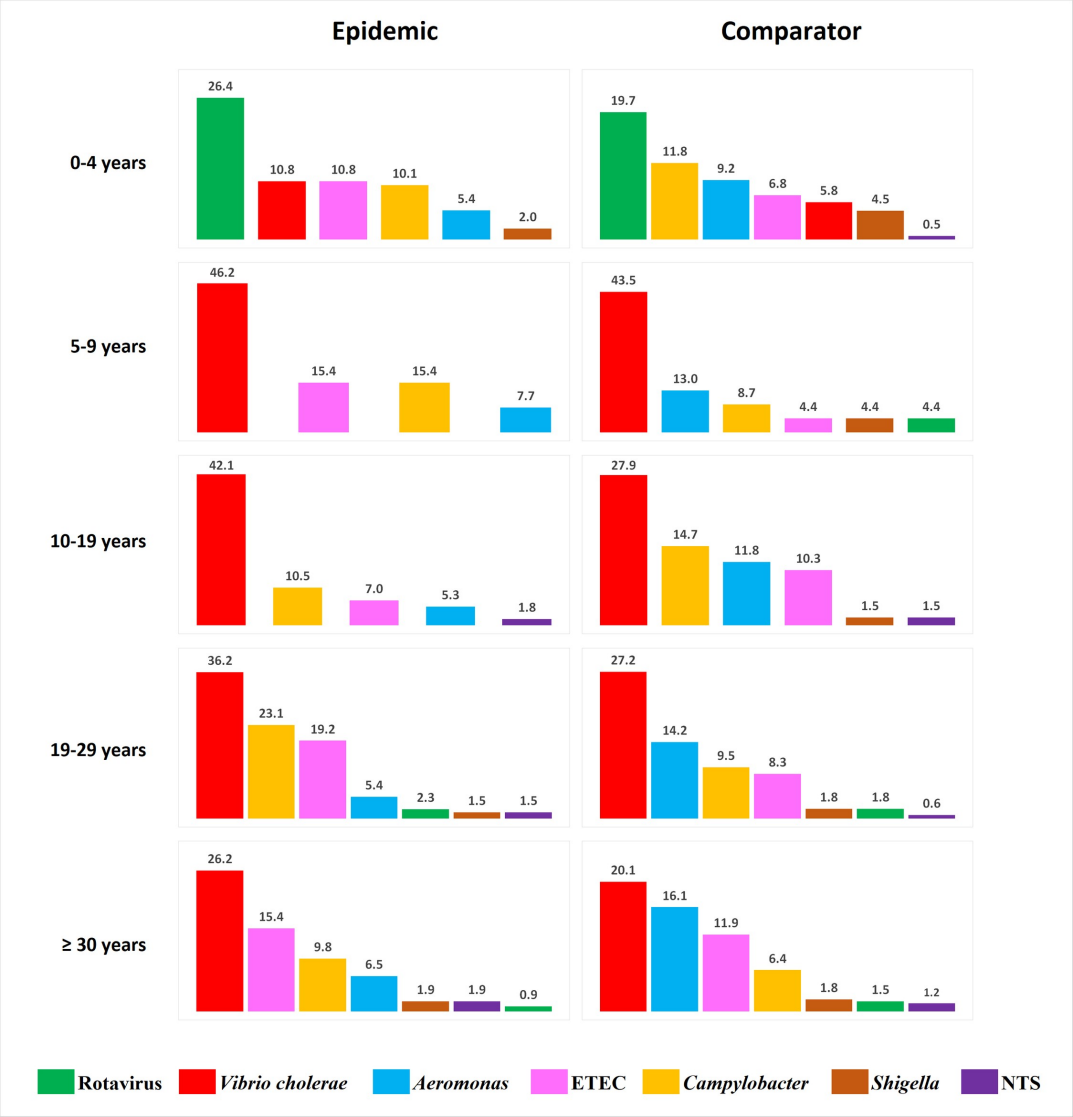
Percent distribution of enteric pathogens isolated from DDSS-enrolled patients treated at icddr,b Dhaka Hospital during the epidemic (cases) and the comparison period (controls) by age groups. The value on top of each column indicates the percentage of samples positive for a particular pathogen. The pathogens (columns) are arranged in decreasing order of prevalence. ETEC, enterotoxigenic *Escherichia coli*; NTS, non-typhoidal *Salmonella*.

Fig 2 shows the percent distribution of the pathogens isolated from the cases and the controls by age groups. *V*. *cholerae* was the most frequently isolated pathogen in all age groups, except in children under five years old. In children younger than five years, rotavirus was the most commonly isolated pathogen, detected in 26% of samples during the epidemic, and 20% of samples during the seasonally matched comparison period.

In unadjusted models, *V*. *cholerae* (OR 1.8, 95% CI: 1.4–2.4; P < 0.001), ETEC (OR 1.7, 95% CI: 1.2–2.3; P = 0.002) and *Campylobacter* (OR 1.4, 95% CI: 1.02–2.0; P = 0.037) were associated with increased odds while *Aeromonas* (OR 0.4, 95% CI: 0.3–0.6; P < 0.001) was associated with reduced odds of diarrhea during the epidemic. The presence of mixed pathogens (coinfection) in stool was associated with higher odds of diarrhea during the epidemic (OR 1.6, 95% CI: 1.1–2.2; P = 0.001). In single-pathogen adjusted models, *V*. *cholerae* (AOR 1.5, 95% CI: 1.1–2.0; P = 0.004), ETEC (AOR 1.5, 95% CI: 1.1–2.2; P = 0.014) and rotavirus (AOR 1.6, 95% CI: 1.03–2.5; P = 0.035) were more likely while *Aeromonas* (AOR 0.4, 95% CI: 0.3–0.6; P < 0.001) was less likely to be associated with diarrhea during the epidemic (Table 2). In dual-pathogen interaction models, the interaction between *Vibrio cholerae* and ETEC (AOR 2.7, 95% CI: 1.3–5.9; P = 0.011) and the interaction between *Vibrio cholerae and Campylobacter* (AOR 2.4, 95% CI: 1.1–5.1; P = 0.020) had statistically significant higher odds of diarrhea during the epidemic (compared to the comparison period) (Table 3).

**Table 3:** Adjusted odds ratio of presenting with diarrhea during the epidemic (compared to the comparison period) for all possible^a^ dual pathogen interaction from multivariable logistic regression models.

Interaction	AOR^b^	95% CI	P
*Vibrio cholerae* × ETEC	2.7	1.3–5.9	0.011
*Vibrio cholerae* × *Campylobacter*	2.4	1.1–5.1	0.020
*Vibrio cholerae* × Rotavirus	2.1	0.4–10.7	0.389
*Vibrio cholerae* × *Shigella*	9.8	0.7–147.4	0.099
ETEC × *Campylobacter*	0.7	0.3–1.6	0.347
ETEC × Rotavirus	1.1	0.3–4.2	0.927
ETEC × *Aeromonas*	0.9	0.3–2.9	0.907
*Campylobacter* × Rotavirus	1.8	0.5–6.5	0.354
*Campylobacter* × *Aeromonas*	0.5	0.1–2.3	0.348
*Campylobacter* × *Shigella*	2.4	0.2–23.9	0.445
Rotavirus × *Aeromonas*	0.5	0.1–4.9	0.577

AOR, adjusted odds ratio; CI, confidence interval; ETEC, enterotoxigenic *Escherichia coli*; NTS, non-typhoidal *Salmonella*.

^a^Following interactions were not examined because coinfection with these combinations were absent among the cases and/or the controls: *Vibrio cholerae* × *Aeromonas*, *Vibrio cholerae* × NTS, ETEC × *Shigella*, ETEC × NTS, *Campylobacter* × NTS, Rotavirus × *Shigella*, Rotavirus × NTS, *Aeromonas* × *Shigella*, *Aeromonas* × NTS, *Shigella* × NTS

^b^Each dual pathogen interaction model is adjusted for the main effect of the stated two pathogens, age, place of residence relative to the location of Dhaka hospital, whether the patient (or their family members) borrowed money or relied on aid to pay for the transport to the hospital, source of drinking water, frequency of collection of water for cooking and washing, and whether the patients or their parents disposed of household solid waste directly outside the house.

**Fig 3:**
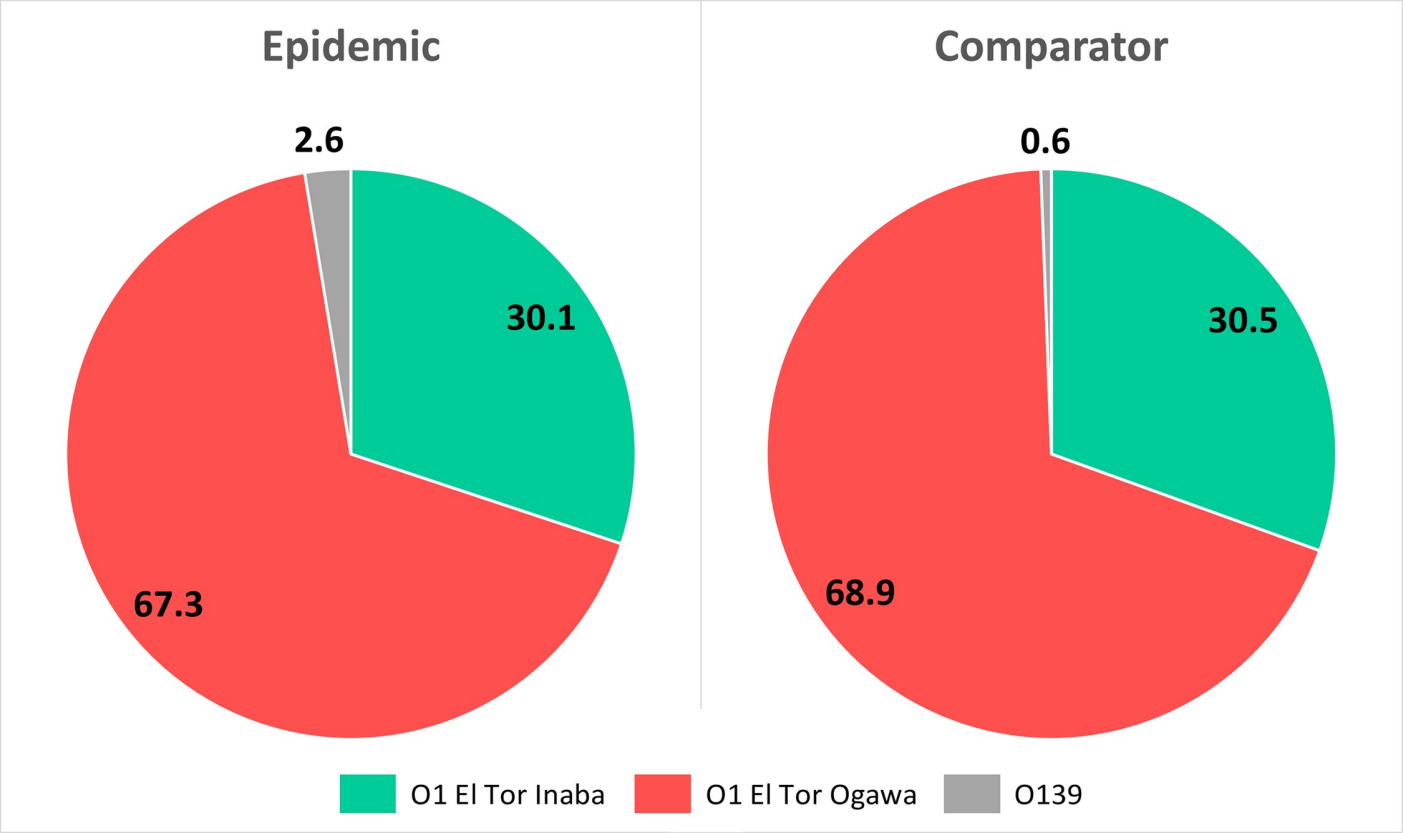
Percent distribution of types of *Vibrio cholerae* isolated from the cases (during the epidemic) and the controls (during the comparison period). No significant difference was found (P = 0.432) in the distribution of *Vibrio cholerae* types between the two groups on Fisher’s exact test.

About 97% and 99% of *V*. *cholerae* isolates belonged to the O1 serogroup and El Tor biotype in the epidemic and the comparison period, respectively. The Ogawa serotype predominated in both periods, accounting for 67% of the isolates among the cases and 69% among the controls. No significant difference was found (P = 0.432) in the distribution of *Vibrio cholerae* types between the cases and the controls (Fig 3).

Resistance pattern of *V*. *cholerae* isolates to azithromycin (P = 0.052), ciprofloxacin (P = 0.478), erythromycin (P = 0.176), and doxycycline (P = 0.604) was comparable between the cases and the seasonally matched controls. Resistance to tetracycline and trimethoprim–sulphamethoxazole was less frequent among the cases compared to the controls (P < 0.001) (Table 4).

**Table 4:** Antimicrobial resistance pattern of *Vibrio cholerae* isolated from the cases (during the epidemic) and the controls (during the comparison period).

Antibiotic^a^	Epidemic (153), n (%)	Comparator (164), n (%)	P
Azithromycin	4 (2.7)	0 (0)	0.052
Ciprofloxacin	1 (0.7)	0 (0)	0.478
Erythromycin	2 (1.7)	0 (0)	0.176
Doxycycline	0 (0)	14 (9.0)	0.604
Tetracycline	3 (2.5)	51 (31.5)	<0.001
Trimethoprim–sulphamethoxazole	106 (71.1)	164 (100)	<0.001

^a^Percentages calculated from non-missing values. Number of missing values: Azithromycin = 6, Ciprofloxacin = 3, Erythromycin = 34, Doxycycline = 148, Tetracycline = 35, and Trimethoprim–sulphamethoxazole = 4.

### Hospital presentation and clinical course

About 99% and 98% of patients presented with watery stools during the epidemic and the comparison period, respectively. Seventy percent of patients had a passage of more than ten stools in the past 24 hours during both periods. On average, the cases presented earlier after the onset of diarrhea than the seasonally matched controls (19 hours vs. 22 hours; P = 0.021). In contrast to the controls, the cases were more likely to have vomiting (OR 1.7, 95% CI: 1.3–2.3; P < 0.001) and less likely to have a fever (OR 0.8, 95% CI: 0.6–0.9; P = 0.015) on admission. While the proportion of patients presenting with some dehydration was comparable between the periods (OR 0.9, 95% CI: 0.7–1.1; P = 0.337), the cases were more likely to be severely dehydrated (OR 1.6, 95% CI: 1.3–2.0; P < 0.001) on admission. Compared to the controls, the cases were more likely to require inpatient admission (OR 2.5, 95% CI: 1.9–3.3; P < 0.001), IV rehydration (OR 1.7, 95% CI: 1.4–2.1; P < 0.001), and antibiotics (OR 2.2, 95% CI: 1.8–2.7; P < 0.001).

The average duration of hospital stays (11 hours vs. 10 hours; P = 0.105) and the odds of death (OR 0.8, 95% CI: 0.4–1.6; P = 0.566) did not significantly differ between the epidemic and the seasonally matched comparison period. Case fatality rates were low in both periods, with 13/29212 (0.04%) in the epidemic and 28/51900 (0.05%) in the comparison period (Table 5). Of the 13 patients who died during the epidemic, ten were under five years old (Table 6). None of the patients died of dehydrating diarrhea alone; all were suffering from one or more of the comorbidities, including severe pneumonia, septic shock, severe acute malnutrition, congenital cyanotic heart disease, and post-chemotherapy complications.

**Table 5:** Hospital presentation and clinical course in DDSS-enrolled patients treated at icddr,b Dhaka Hospital during the epidemic (cases) compared to the patients treated during the comparison period (controls).

Presentation and clinical course	Cases (562), n (%)	Controls (969), n (%)	OR^a^	95% CI	P
**History**					
Nocturnal (7pm-7am) onset of diarrhea	258 (45.9)	418 (43.1)	1.1	0.9–1.4	0.293
Duration of diarrhea before coming to Dhaka Hospital, hours; median (IQR)	19 (10, 38)	22 (11, 46)	-	-	0.021
Frequency of stool, >10 times in past 24 hours	392 (69.8)	673 (69.5)	1.0	0.8–1.3	0.903
Home use of ORS	462 (82.2)	762 (78.6)	1.3	1.0–1.6	0.093
Home use of other oral medications^b^	408 (72.6)	679 (70.1)	1.1	0.9–1.4	0.294
**On admission**					
Watery stool	558 (99.3)	945 (97.5)	3.5	1.2–10.3	0.020
Blood in stool	5 (0.9)	20 (2.1)	0.4	0.2–1.1	0.090
Abdominal pain	369 (65.7)	649 (67.0)	0.9	0.8–1.2	0.599
Vomiting	479 (85.2)	744 (76.8)	1.7	1.3–2.3	<0.001
Fever (>37.8°C)	197 (35.1)	401 (41.4)	0.8	0.6–0.9	0.015
Some dehydration	162 (28.2)	302 (31.2)	0.9	0.7–1.1	0.337
Severe dehydration	301 (53.6)	403 (41.6)	1.6	1.3–2.0	<0.001
**Clinical course**					
Inpatient admission required at Dhaka Hospital	481 (85.6)	683 (70.5)	2.5	1.9–3.3	<0.001
IV fluid required for rehydration at Dhaka Hospital	333 (59.3)	450 (46.4)	1.7	1.4–2.1	<0.001
Antibiotics prescribed at Dhaka Hospital	394 (70.1)	500 (51.6)	2.2	1.8–2.7	<0.001
Duration of hospital stay, hours; median (IQR)	11 (5, 20)	10 (4, 21)	-	-	0.105
Death^c^	13/29212 (0.04)	28/51900 (0.05)	0.8	0.4–1.6	0.566

OR, odds ratio; CI, confidence interval; IQR, interquartile range; ORS, oral rehydration solution; IV, intravenous.

^a^Odds ratio of the clinical features among the cases compared to the controls estimated from a simple binomial logistic regression model.

^b^Other oral medications included mostly zinc, ondansetron, ranitidine, loperamide, paracetamol, and antibiotics.

^c^Figures for hospital patient visits and in-hospital deaths were obtained from the hospital registry.

**Table 6:** Estimated deaths averted by taking care of patients at icddr,b Dhaka Hospital during the 2018 diarrhea epidemic (N = 29212).

Age group	Number of patients	Number of deaths	Number of deaths averted		Percentage of potential deaths averted		Percentage of patients that would have died without the hospital care	
-	-	-	Lower bound^a^	Upper bound^b^	Lower bound	Upper bound	Lower bound	Upper bound
0–4 years	7692	10	837	1411	98.8	99.3	10.9	18.3
5–9 years	675	0	374	520	100	100	55.4	77.0
10–19 years	2963	0	1789	2288	100	100	60.4	77.2
20–29 years	6757	0	3826	5146	100	100	56.6	76.2
≥ 30 years	11125	3	5697	7900	99.94	99.96	51.2	71.0
Total	29212	13	12523	17265	99.90	99.92	42.9	59.1

^a^The minimum estimate (lower bound) of deaths averted was composed of 80% of all patients who survived after presenting with severe dehydration.

^b^The maximum estimate (upper bound) of deaths averted was composed of all patients who survived after presenting with severe dehydration, or with some or no dehydration but required intravenous rehydration later.

**Fig 4:**
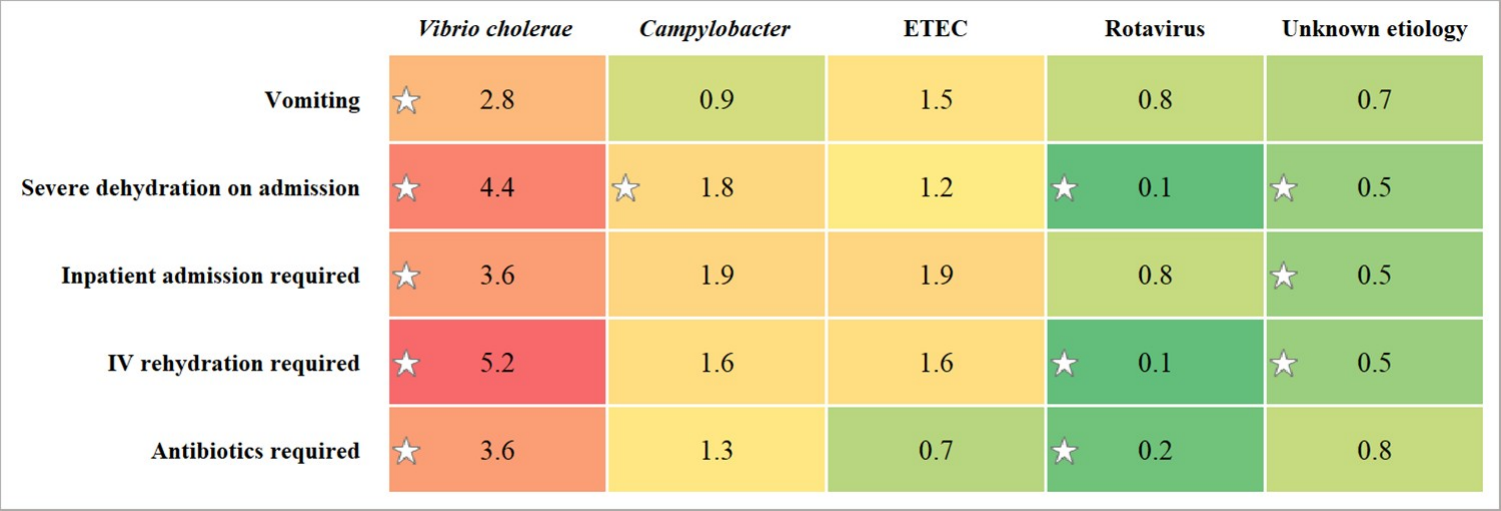
Relative effect of selective enteric pathogens on disease severity during the epidemic. The value in each cell is the age-adjusted odds ratio of a particular attribute of disease severity for a specific pathogen. The cells are coded using a red-yellow-green color gradient. The red end of the spectrum represents increased odds, and the green end represents decreased odds. The darker the shade, the stronger the effect. Asterisk indicates a statistically significant (P < 0.05) odds ratio. The age-adjusted odds ratios and the corresponding P values were obtained from logistic regression models. To simplify the analysis, coinfections were ignored in the models. ETEC, enterotoxigenic *Escherichia coli*.

Fig 4 shows the relative effect of selective enteric pathogens on disease severity during the epidemic (among the cases). Cholera was found to cause the most severe illnesses during the epidemic, significantly increasing the odds of vomiting (AOR 2.8, 95% CI: 1.5–5.5; P = 0.002), severe dehydration on admission (AOR 4.4, 95% CI: 2.8–6.9; P < 0.001), and requiring inpatient admission (AOR 3.6, 95% CI: 1.4–8.9; P = 0.006), IV rehydration (AOR 5.2, 95% CI: 3.2–8.5; P < 0.001) and antibiotics (AOR 3.6, 95% CI: 2.1–6.3; P < 0.001) (Fig 4 and S4 Table).

### Deaths averted

As described above, the minimum and maximum figures for the potential diarrhea-related mortality averted during the epidemic by icddr,b Dhaka Hospital was estimated based on the number of patients who presented with severe dehydration or required IV fluids for rehydration. According to our estimate, Dhaka Hospital averted between 12,523 (minimum) and 17,265 (maximum) deaths by taking care of 29,212 patients during the diarrhea epidemic in 2018. The hospital managed to avoid 99.9% of potential diarrhea-related deaths. Overall, an estimated 43–59% of patients would have died in the absence of the service rendered by the hospital. The percentage of patients that would have expired without hospital care was highest among adolescents (60–77%) and lowest among children younger than five years (11–18%) (Table 6).

## Discussion

To our knowledge, this is the first study that investigated the putative causes of the diarrhea epidemic in Dhaka and adjoining areas in 2018, examined the clinical presentation and outcome of the patients, and estimated the deaths averted by treating patients at icddr,b Dhaka Hospital during the epidemic. Our results suggest that *V*. *cholerae* was the primary pathogen responsible for the 2018 diarrhea epidemic, considering its high isolation rate (27%) and association with diarrhea (AOR 1.5, P = 0.004) and disease severity (vomiting: AOR 2.8, P = 0.002 and severe dehydration: AOR 4.4, P < 0.001) during the epidemic among patients treated at Dhaka Hospital. The isolation rate of *V*. *cholerae* increased to 27% during the epidemic compared to 17% during the seasonally matched comparison period. This increase from the baseline frequency in the proportion of *V*. *cholerae* isolated from patients visiting Dhaka Hospital was also observed during the earlier flood-related diarrhea epidemics in Bangladesh in 1998 (42% from 20%) and 2004 (23% from 11%) [14].

The types of *V*. *cholerae* responsible for the 2018 epidemic were comparable to the organisms causing cholera during the comparison period (P = 0.432). Essentially all (97% and 99%) *V*. *cholerae* isolates were of O1 serogroup and El Tor biotype, and Ogawa was the predominating (67% and 69%) serotype during both the epidemic and the comparison period. Similar to the 2018 epidemic, the distribution of *V*. *cholerae* serotype during the earlier flood-associated diarrhea epidemics in the country was comparable with that of the preceding months [14]. Schwartz et al. conjectured that the host-mediated amplification of circulating strains of *V*. *cholerae*, not the emergence of new environmental isolates, might account for these epidemics [14]. During the 2018 epidemic, patients were more likely to present with severe dehydration (OR 1.7, P < 0.001) and vomiting (OR 1.6, P < 0.001), mostly attributed to *V*. *cholerae*. Harris et al. have shown that the severity of dehydration in cholera patients has increased since the beginning of this millennium [15]. The emergence of a new variant of *V*. *cholerae* O1 El Tor that secretes classical-type cholera toxin could underlie this increase in disease severity. Since 2002, this variant biotype has virtually displaced typical *V*. *cholerae* O1 El Tor in Bangladesh [5].

The *V*. *cholerae* isolates from the epidemic were more frequently susceptible to tetracycline (P < 0.001) and trimethoprim–sulphamethoxazole (P < 0.001) than the isolates from the comparison period. The short-term reappearance of sensitivity to these antimicrobials during the epidemic is due to poorly understood environmental and bacterial factors. However, it underscores the importance of routine antimicrobial susceptibility tests for *V*. *cholerae* in treatment intervention.

Our results suggest that ETEC (14% isolation), *Campylobacter* (13% isolation), and rotavirus (8% isolation) played a secondary role in the 2018 diarrhea epidemic. In single-pathogen models, ETEC (AOR 1.5, P = 0.014) and rotavirus (AOR 1.6, P = 0.035) were associated with higher odds of diarrhea during the epidemic. Statistically significant interaction of ETEC (AOR 2.7, P = 0.011) and *Campylobacter* (AOR 2.4, P = 0.020) with *Vibrio cholerae* indicates that coinfection of these combinations further increased the odds of diarrhea during the epidemic. Apart from the 2018 epidemic, ETEC played a major role in a flood-related diarrhea epidemic in Bangladesh during the summer of 2004, isolated from 18% of patients presenting to Dhaka Hospital during the epidemic [42]. Although the average number of patients visiting Dhaka Hospital (mean cases per day) with *Campylobacter*-associated diarrhea increased from the baseline (78 from 33, P < 0.001) during the 1998 epidemic, *Campylobacter* never appeared to be a key pathogen in any diarrhea epidemics before 2018 [14,15,43]. On the contrary, our results revealed the epidemic potential of *Campylobacter*. During the 2018 epidemic, rotavirus infection was prevalent (26%) only among children younger than five years, as in earlier epidemics [15].

Our results suggest that those who were adolescents and adults (age ≥10 years), residents of the Dhaka metropolitan area (within a 10 km radius of Dhaka Hospital), relatively poor (reliant on aid or borrowing money to pay for the transport to the hospital), and reported unsafe WASH practices (use of tap water for drinking, low frequency of water collection indicating storage of water for a long duration, and disposal of the solid waste directly outside the house) were more likely to present with diarrhea during the epidemic. These findings are consistent with previous studies investigating the association of sociodemographic factors with flood-associated diarrhea and non-flood cholera in Bangladesh [44–47].

Poor WASH practices are closely related to low socioeconomic status [48]. The excess of infection during the epidemic might have resulted from the persistence of poor WASH practices, particularly in low-income communities. In the metropolitan area of Dhaka, tap water is the main source of drinking water compared to tubewell water in many places outside Dhaka. The association of drinking tap water with the epidemic might be related to the visit of an increased number of patients from the metropolitan area during the epidemic. The transmission of enteric pathogens through drinking untreated tap water is a likely explanation since microbiological contamination of supply water is not uncommon in neighborhoods of Dhaka city [49,50]. Moreover, Adolescents and adults, the working-age population in low-income communities, tend to consume unhygienic street foods and drinks, such as iced lemon sherbet and sugarcane juice, during the day-long working hours in summer. These foods and drinks are potential sources of diarrheagenic pathogens [51].

Our findings have implications for public health policies and programs to prevent enteric infections and diarrhea in low-income communities. Efforts should be reevaluated and increased on improving water quality and delivery systems, sanitation infrastructure, personal and food hygiene, and household waste management. Although expensive, the development and blanket deployment of vaccines against the most common diarrheagenic pathogens in low-income communities can be another option.

Despite the marked increase in the absolute number of patient visits, disease severity, inpatient admissions (OR 2.5, P < 0.001), and the requirement of medical supplies, including IV fluids (OR 1.7, P < 0.001) and antibiotics (OR 2.2, P < 0.001), there was no significant increase in the average duration of hospital stay (11 hours vs. 10 hours, P = 0.105) or case fatality (OR 0.8, P = 0.566) during the epidemic. Of the 29,212 patients the hospital treated in less than six weeks during the epidemic, only 13 died. The patients seemed to respond effectively to the management protocol, including the use of replacement fluids and antibiotics. By taking care of the epidemic, the hospital avoided between 12,523 and 17,265 unnecessary deaths. In the absence of the care provided by the hospital, 43–59% of the patients presented would have died.

The low case fatality rate during the epidemic, in spite of the increased disease severity and patient load, is an indication of excellent clinical management at the hospital. Without this hospital, patients would have had to visit other health facilities with little experience of dealing with such large epidemics of diarrhea, ultimately leading to the loss of many lives. Many patients would have ended up receiving care at private hospitals, costing this low-income group a fortune. During the 2018 epidemic, icddr,b Dhaka Hospital saved several thousand precious lives without the patients spending a cent.

The strengths of this study are that we had an unbiased systematic sample and high-quality lab results, obtained the actual number of patient visits and the list of deaths from the hospital registry, and analyzed a reasonably large dataset. However, the findings of the study should be interpreted in the context of the limitations of the study design and available data. An important limitation of the study is that the comparison group is comprised of seasonally matched hospital controls rather than concurrent community controls. Institution-based data may fall short of adequately representing the patient population in the community at large. However, the exclusivity of icddr,b Dhaka Hospital as a diarrhea treatment facility and people’s preference for the hospital to seek care for diarrhea (hence, the colloquial name, Cholera Hospital), the steadiness of the hospital management protocol over the study period, the large sample size, and the comprehensive analytical approach adopted might compensate for the limitations for the most part.

It is important to realize that no pathogens were identified in 49% of stool samples during the epidemic. The lower disease severity in rotaviral diarrhea compared to diarrhea of unknown etiology indicated that some undetected pathogens might be responsible for the cases of diarrhea with unknown etiology. The unidentified pathogens might be any of the organisms causing secretory diarrhea, which the DDSS did not screen for, such as norovirus, adenovirus, enteropathogenic *E*. *coli*, or *Entamoeba histolytica* [2,4]. Another limitation was that we did not evaluate the effect of microbiological factors, including the presence of toxigenic environmental isolates and lytic bacteriophages, host factors such as the level of population-level immunity, and meteorological factors, such as temperature, rainfall, surface water salinity, sea level, and El Nino Southern Oscillation activity, which may influence the occurrence of endemic and epidemic diarrhea [52–55].

To calculate the number of deaths averted, we followed the methods described by Oberle et al. [40] with some necessary adaptation. We differed mainly as to how the level of dehydration was assessed. Oberle et al. quantified the dehydration percentage based on the admission and discharge body weights and stool volumes, while we used the ‘Dhaka Method’ [5] to assess dehydration clinically. The method estimated the mortality averted that were attributed to rehydration therapy but did not take into account the contribution of antibiotics, which might have lowered the volume of stool and shortened the duration of diarrhea in these patients [40]. Although 70% of patients received antimicrobial therapy during the epidemic, ours is a realistic estimate because antibiotic therapy is an integral component of bacterial diarrhea management.

In conclusion, our data demonstrated that *V*. *cholerae* played the primary role in the 2018 diarrhea epidemic in Dhaka and adjoining areas. *Campylobacter*, ETEC, and rotavirus also contributed to the etiologic profile. Those who were residents of the metropolitan area, relatively poor, lacked safe and appropriate WASH practices, and adolescents and adults were the most vulnerable groups. Dhaka Hospital successfully dealt with the huge patient load during the epidemic, nearly avoiding all the potential deaths. Patients, most of whom presented with watery diarrhea and vomiting, seemed to respond well to rehydration therapy and antibiotics. During the epidemic, the hospital saved between 12,523 and 17,265 lives. An estimated 43–59% of the patients would have died had the hospital not provided good clinical care.

## Supporting information

S1 Table Key variables available in DDSS database. (PDF)

S2 Table Characteristics of DDSS-enrolled patients visiting Dhaka Hospital during the comparison period in 2017 (N = 402) and in 2019 (N = 567). (PDF)

S3 Table Clinical presentation of culture-confirmed cholera patients presenting during the epidemic (N = 153) compared to the comparison period (N = 164). (PDF)

S4 Table Effect of selective enteric pathogens on disease severity during the epidemic. (PDF)

S1 Fig Relative prevalence of enteric pathogens during the epidemic versus the comparison period across age groups. The epidemic and the comparison period are color-coded; the color of the period, which had a higher prevalence of a certain pathogen between the two, is displayed. Chi-square or Fisher’s exact test, as appropriate, was used to compare the proportion of a pathogen between the two periods. The significance in difference (P value) is indicated by ***** for <0.001, **** for <0.01, *** for <0.05, ** for <0.1, and * for <0.2. ETEC, enterotoxigenic Escherichia coli; NTS, non-typhoidal Salmonella.
(TIF)
