# Supplementary material for: Taking care of a diarrhea epidemic in an urban hospital in Bangladesh: Appraisal of putative causes, presentation, management, and deaths averted
Source: PLoS Negl Trop Dis. 2021 Nov 15;15(11):e0009953. doi: 10.1371/journal.pntd.0009953 (PMC8629377; doi:10.1371/journal.pntd.0009953)
Supplement: S1 Table — (PDF) [file pntd.0009953.s001.pdf]

S1 Table. Key variables available in DDSS database.

Sl No	Variables available	Included in the analysis?
1	Age	Yes
2	Sex	Yes
3	Religion	No
4	Number of family members	Yes
5	Place of residence	Yes
6	Highest education in the family	Yes
7	Monthly family income	Yes
8	Variables indicating possession of household assets	Yes
9	Variables indicating housing condition	Yes
10	Father's/mother's age (if patient's age less than 15 years)	No
11	Primary occupation of father/mother (if patient's age less than 15 years)	No
12	Father/mother member of a cooperative/NGO (if patient's age less than 15 years)	No
13	Father/mother reads newspaper (if patient's age less than 15 years)	No
14	Father/mother watches TV (if patient's age less than 15 years)	No
15	Father/mother listens to radio (if patient's age less than 15 years)	No
16	Father/mother smokes (if patient's age less than 15 years)	No
17	Patient smokes (if patient's age more than 15 years)	No
18	Source of drinking water	Yes
19	Any treatment of drinking water	Yes
20	Source of water for washing	Yes
21	Distance of main source of water for drinking/cooking/washing from kitchen	No
22	Frequency of water collection for drinking	Yes
23	Frequency of water collection for cooking	Yes
24	Frequency of water collection for washing	Yes
25	Type of toilet	Yes
26	Mode of disposal of solid waste	Yes
27	Weight at admission	No
28	Weight at discharge	Yes
29	Recumbent length/Standing height	Yes
30	MUAC (for children under 5 years)	No
31	Birth order (for children under 5 years)	No
32	Feeding practices (for children up to 3 years)	No
33	When took vitamin A capsule (for children under 5 years)	Yes
34	History of measles (for children under 5 years)	Yes

35	Any diarrhea of the child other than this episode in last 7/14/28 days	No
36	Any cough/fever/sneezing/runny nose/rapid respiration/breathing difficulty/ear discharge/husky voice of the child in last 14 days	No
37	Immunization history (for children under 5 years)	No
38	History of diarrhea of any family members in the past week	Yes
39	Number of deaths in the last five years from diarrhea	No
40	Time of onset of diarrhea	Yes
41	Duration of diarrhea before admission	Yes
42	Number of stools in past 24 hours	Yes
43	Character of stool	Yes
44	Abdominal pain	Yes
45	Vomiting	Yes
46	Variables related to physical examination findings	Yes
47	Home use of ORS	Yes
48	Home use of other oral medications	Yes
49	Managed in in-patient/OPD	Yes
50	Rehydration method (ORS/IV)	Yes
51	Use of antibiotics at Dhaka hospital	Yes
52	Duration of hospital stay	Yes
53	In-hospital death	Yes
54	How much money spent for traveling from home to Dhaka Hospital	No
55	Source of the transport cost	Yes
56	Time required to reach Dhaka Hospital	No
57	How much money paid to private clinics/physicians before coming to Dhaka Hospital	No
58	Presence of *Vibrio cholerae* in stool sample	Yes
59	Type of *Vibrio cholerae*	Yes
60	Presence of enterotoxigenic *Escherichia coli* in stool sample	Yes
61	Presence of *Campylobacter* in stool sample	Yes
62	Presence of *Aeromonas* in stool sample	Yes
63	Presence of *Shigella* in stool sample	Yes
64	Presence of Typhoidal *Salmonella* in stool sample	No
65	Presence of Non-typhoidal *Salmonella* in stool sample	Yes
66	Presence of Rotavirus in stool sample	Yes
67	Antimicrobial resistance pattern of *Vibrio cholerae* isolates	Yes
68	Antimicrobial resistance pattern of *Shigella* isolates	No
69	Antimicrobial resistance pattern of *Salmonella* isolates	No
