# Supplementary material for: Taking care of a diarrhea epidemic in an urban hospital in Bangladesh: Appraisal of putative causes, presentation, management, and deaths averted
Source: PLoS Negl Trop Dis. 2021 Nov 15;15(11):e0009953. doi: 10.1371/journal.pntd.0009953 (PMC8629377; doi:10.1371/journal.pntd.0009953)
Supplement: S2 Table — (PDF) [file pntd.0009953.s002.pdf]

S2 Table. Characteristics of DDSS-enrolled patients treated at icddr,b Dhaka Hospital during the seasonally matched control periods in 2017 and in 2019.

Characteristics	Aggregate comparison period (969), n (%)	Control period in 2017 (402), n (%)	Control period in 2019 (567), n (%)	P[a]
**Demographic characteristics**				
Age, years				<0.001
0-4	380 (39.2)	193 (48.0)	187 (33.0)	
5-9	23 (2.4)	11 (2.7)	12 (2.1)	
10-19	68 (7.0)	18 (4.5)	50 (8.8)	
20-29	169 (17.4)	57 (14.2)	112 (19.8)	
≥30	329 (34.0)	123 (30.6)	206 (36.3)	
Sex, male	551 (56.9)	239 (40.6)	312 (45.0)	0.170
Family members				0.825
1-4	483 (49.9)	201 (50.0)	282 (49.7)	
5-6	310 (32.0)	125 (31.1)	185 (32.6)	
≥7	176 (18.2)	76 (18.9)	100 (17.6)	
Place of residence[b]				0.475
within 10 km radius of Dhaka Hospital	235 (24.3)	102 (25.4)	133 (23.5)	
within 10-20 km radius of Dhaka Hospital	420 (43.3)	165 (41.0)	255 (45.0)	
outside 20 km radius of Dhaka Hospital	314 (32.4)	135 (33.6)	179 (31.6)	
**Socioeconomic status**				
Highest education in the family, years				0.335
none	207 (21.4)	80 (19.9)	127 (22.4)	
1-5	184 (19.0)	75 (18.7)	109 (19.2)	
6-10	281 (29.0)	131 (32.6)	150 (26.5)	
11-12	174 (18.0)	69 (17.2)	105 (18.5)	
>12	123 (12.7)	47 (11.7)	76 (13.4)	
Family income, below USD 100 per month	137 (14.1)	49 (12.2)	88 (15.5)	0.143
Wealth tertile				0.833
low	332 (34.3)	139 (34.6)	193 (34.0)	
middle	326 (33.6)	131 (32.6)	195 (34.4)	
high	311 (32.1)	132 (32.8)	179 (31.6)	
Reliance on aid or borrowing money for transport cost	190 (19.6)	95 (23.6)	95 (16.8)	0.008
**WASH behavior**				
Source of drinking water, tap water	549 (56.7)	285 (70.9)	264 (46.6)	<0.001
Source of water for washing, tap water	558 (57.6)	288 (71.6)	270 (47.6)	<0.001
Frequency of water collection for drinking, ≤2 times per day	438 (45.2)	189 (47.0)	249 (43.9)	0.339
Frequency of water collection for cooking, ≤2 times per day	720 (74.3)	303 (75.4)	417 (73.5)	0.521
Frequency of water collection for washing, ≤2 times per day	82 (8.5)	72 (17.9)	10 (1.8)	<0.001

Drinking untreated water	547 (56.5)	224 (55.7)	323 (57.0)	0.700
Use of sanitary toilet	12 (1.2)	5 (1.2)	7 (1.2)	0.990
Disposal of solid waste directly outside the house	736 (76.0)	347 (86.3)	389 (68.6)	<0.001
**Nutrition and health**				
Nutritional status[c]				0.237
thin	173 (19.4)	72 (19.3)	101 (19.4)	
normal	611 (68.3)	247 (66.2)	364 (69.9)	
overweight	110 (12.3)	54 (14.5)	56 (10.8)	
Stunted[c] (aged 0-19 years)	83 (18.0)	41 (19.1)	42 (17.1)	0.592
Took vitamin A capsule in past 3 months (aged 0-4 years)	181 (47.6)	75 (38.9)	106 (56.7)	0.001
Recent measles (aged 0-4 years)	22 (5.8)	10 (5.2)	12 (6.4)	0.606
Family members had diarrhea in the past week	122 (12.6)	48 (11.9)	74 (13.1)	0.608

[a]P values obtained from chi-square tests comparing proportions between 2017 and 2019.

[b]Place of residence relative to the location of Dhaka Hospital

[c]Percentages calculated from non-missing values. Number of missing values: Nutritional status = 75, Stunted = 11.
