# Supplementary material for: Taking care of a diarrhea epidemic in an urban hospital in Bangladesh: Appraisal of putative causes, presentation, management, and deaths averted
Source: PLoS Negl Trop Dis. 2021 Nov 15;15(11):e0009953. doi: 10.1371/journal.pntd.0009953 (PMC8629377; doi:10.1371/journal.pntd.0009953)
Supplement: S3 Table — (PDF) [file pntd.0009953.s003.pdf]

S3 Table. Clinical presentation of culture-confirmed cholera patients treated at icddr,b Dhaka Hospital during the epidemic compared to the comparison period.

**Presentation**	**Epidemic (153), n (%)**	**Comparator (164), n (%)**	**OR (95% CI)[a]**	**P[a]**
Frequency of stool, >10 times in past 24 hours	117 (76.5)	123 (75.0)	1.1 (0.6-1.8)	0.760
Abdominal pain	101 (66.0)	110 (67.1)	1.0 (0.6-1.5)	0.842
Vomiting	142 (92.8)	142 (86.6)	2.0 (0.9-4.3)	0.074
Fever (>37.8°C)	29 (19.0)	47 (28.7)	0.6 (0.3, 0.99)	0.044
Some dehydration	26 (17.0)	32 (19.5)	0.8 (0.5-1.5)	0.562
Severe dehydration	120 (78.4)	125 (76.2)	1.1 (0.7-1.9)	0.639

OR, odds ratio; CI, confidence interval.

[a]Odds ratio (and 95% CI and P value) of the clinical features during the epidemic compared to the comparison period estimated from a simple binomial logistic regression model.
