# Supplementary material for: Taking care of a diarrhea epidemic in an urban hospital in Bangladesh: Appraisal of putative causes, presentation, management, and deaths averted
Source: PLoS Negl Trop Dis. 2021 Nov 15;15(11):e0009953. doi: 10.1371/journal.pntd.0009953 (PMC8629377; doi:10.1371/journal.pntd.0009953)
Supplement: S4 Table — (PDF) [file pntd.0009953.s004.pdf]

S4 Table. Effect of selective enteric pathogens on disease severity during the epidemic.

Pathogen	Vomiting	Severe dehydration on admission	Inpatient admission required	IV rehydration required	Antibiotics required
	**OR (95% CI); P[a]**	**OR (95% CI); P[a]**	**OR (95% CI); P[a]**	**OR (95% CI); P[a]**	**OR (95% CI); P[a]**
Vibrio cholerae	2.8 (1.5, 5.5); 0.002	4.4 (2.8, 6.9); <0.001	3.6 (1.4, 8.9); 0.006	5.2 (3.2, 8.5); <0.001	3.6 (2.1, 6.3); <0.001
Campylobacter	0.9 (0.4, 1.7); 0.685	1.8 (1.1, 3.1); 0.025	1.9 (0.7, 5.0); 0.175	1.6 (0.9, 2.7); 0.089	1.3 (0.7, 2.4); 0.389
ETEC	1.5 (0.7, 3.1); 0.310	1.2 (0.7, 1.9); 0.525	1.9 (0.7, 5.0); 0.188	1.6 (0.9, 2.7); 0.082	0.7 (0.4, 1.3); 0.256
Rotavirus	0.8 (0.3, 1.9); 0.555	0.1 (0.03, 0.3); <0.001	0.8 (0.4, 1.7); 0.650	0.1 (0.02, 0.2); <0.001	0.2 (0.1, 0.4); <0.001
Diarrhea of unknown etiology	0.7 (0.4, 1.1); 0.151	0.5 (0.4, 0.7); <0.001	0.5 (0.3, 0.9); 0.022	0.5 (0.4, 0.7); <0.001	0.8 (0.6, 1.3); 0.423

OR, odds ratio; CI, confidence interval; ETEC, enterotoxigenic *Escherichia coli*.

[a]Odds ratio (and 95% CI and P value) of the attributes indicating disease severity for the enteropathogens were estimated from binomial logistic regression models, adjusted for age.
