# Supplementary figures and images for: Taking care of a diarrhea epidemic in an urban hospital in Bangladesh: Appraisal of putative causes, presentation, management, and deaths averted
Source: PLoS Negl Trop Dis. 2021 Nov 15;15(11):e0009953. doi: 10.1371/journal.pntd.0009953 (PMC8629377; doi:10.1371/journal.pntd.0009953)

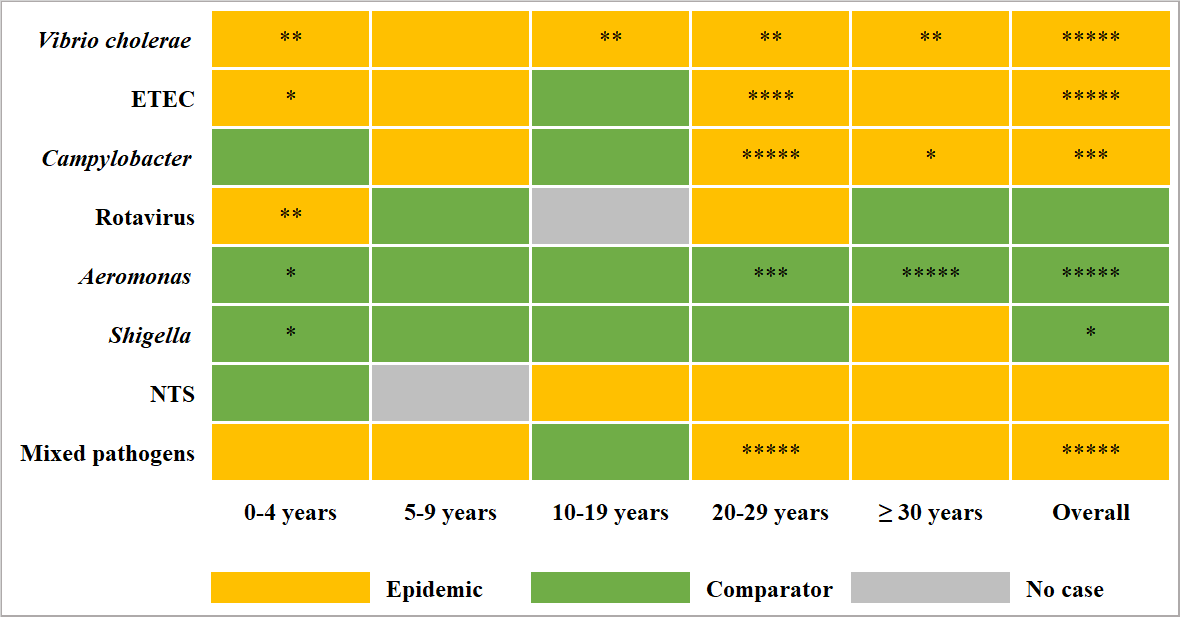

Supplement: S1 Fig — The epidemic and the comparison period are color-coded; the color of the period, which had a higher prevalence of a certain pathogen between the two, is displayed. Chi-square or Fisher’s exact test, as appropriate, was used to compare the proportion of a pathogen between the two periods. The significance in difference (P value) is indicated by ***** for <0.001, **** for <0.01, *** for <0.05, ** for <0.1, and * for <0.2. ETEC, enterotoxigenic Escherichia coli; NTS, non-typhoidal Salmonella. (TIF) [file pntd.0009953.s005.tif]
